# *NR1H4* mutation and rapid progressive intrahepatic cholestasis in infancy: A case report and literature review

**DOI:** 10.1002/ccr3.8531

**Published:** 2024-02-23

**Authors:** Chiao‐Yu Yang, Hung‐Wen Tsai, Yen‐Yin Chou, Yao‐Jong Yang

**Affiliations:** ^1^ Department of Pediatrics National Cheng Kung University Hospital Tainan Taiwan; ^2^ Department of Pediatrics, An Nan Hospital China Medical University Tainan Taiwan; ^3^ Department of Pathology National Cheng Kung University Hospital Tainan Taiwan; ^4^ Institute of Clinical Medicine College of Medicine, National Cheng Kung University Tainan Taiwan

**Keywords:** neonatal cholestasis, *NR1H4* gene, PFIC‐5, shear wave elastography

## Abstract

Farnesoid X receptor (FXR) is a nuclear bile acid receptor encoded by the *NR1H4* gene, a vital regulator of bile acid homeostasis. Pathogenic mutations of *NR1H4* manifest as low gamma‐glutamyl transferase (GGT) cholestasis with rapid progression to liver failure, which is referred to as progressive familial intrahepatic cholestasis 5 (PFIC‐5). Herein, we present a case with rapid progressive cholestasis, liver failure in early infancy with the *NR1H4* termination mutation.

## 1 INTRODUCTION

Neonatal cholestasis is a challenging condition for clinicians to identify the underlying cause. Recently, genetic testing to detect the etiology of cholestasis by next‐generation sequencing (NGS) has been proposed.^1^ It helps clinicians to rapidly diagnose and predict the prognosis of patients with progressive cholestatic diseases.

The *NR1H4* gene is located on chromosome 12q23 and encodes a bile acid nuclear receptor called farnesoid X receptor (FXR), a key regulator of bile acid metabolism by binding to promoter regions of target genes.^2^ FXR promotes bile acid secretion by upregulating canalicular transporters such as the bile salt export pump (BSEP) and multidrug resistance protein 3 (MDR3) receptors. FXR also activates transcription of the small heterodimer partner (SHP) gene to repress bile acid synthesis by inhibiting the expression of cholesterol 7‐alpha hydroxylase (CYP7A1), a rate‐limiting enzyme for bile acid synthesis.^2^ Bile acid activated FXR induces the expression of intestinal fibroblast growth factor 19 (FGF19) which acts in the liver to suppress bile acid synthesis. Thus *NR1H4* mutation causes down regulation of FXR that affects bile acid metabolism leading to intrahepatic cholestasis.

*NR1H4* mutations are associated with different types and severity of cholestasis including drug induced cholestasis, intrahepatic cholestasis of pregnancy^3^, ^4^ and neonatal cholestasis which is also called progressive familial intrahepatic cholestasis 5(PFIC‐5). PFIC‐5 is characterized by low to normal gamma‐glutamyl transferase (GGT) cholestasis, vitamin K‐refractory coagulopathy, and rapid progression to liver failure.^5^ Here, we present a patient with characteristic clinical and laboratory manifestations of PFIC‐5 and the *NR1H4* mutation.

## 2 CASE PRESENTATION

### 2.1 Case history and examination

A female baby was delivered at 39 weeks and 4 days of gestation after an uneventful pregnancy. Jaundice was noted when she was 2 months old and visiting for vaccinations. On physical examination, she was well‐nourished but had icterus and hepatomegaly. Blood tests revealed direct hyperbilirubinemia with total and direct bilirubin of 18.8/10.2 mg/dL, aspartate transaminase (AST)/alanine transaminase (ALT) of 535/323 U/L, GGT of 33 U/L, and prolonged international normalized ratio (INR) of up to 2.2 (Table 1).

**TABLE 1:** Laboratory results on different age.

Age of patient (month)	Total bilirubin (mg/dL)	Direct bilirubin (mg/dL)	AST (U/L)	ALT (U/L)	PT (INR)	aPTT (sec)	GGT (U/L)	AFP (ng/mL)	Albumin (g/dL)	Ammonia (μg/dL)
2	17	13.1	455	323	2	59.7	33		3.7	104
4	8.5	7.1	250	222	1.6	54.2			4.6	
7	20.6	18.1	538	374	2.6	67.9			3.5	
9	38.9	19.9	213	111	4.58	83.7	16	>80,000	2.44	385

### 2.2 Differential diagnosis, investigations, and treatment

**FIGURE 1:**
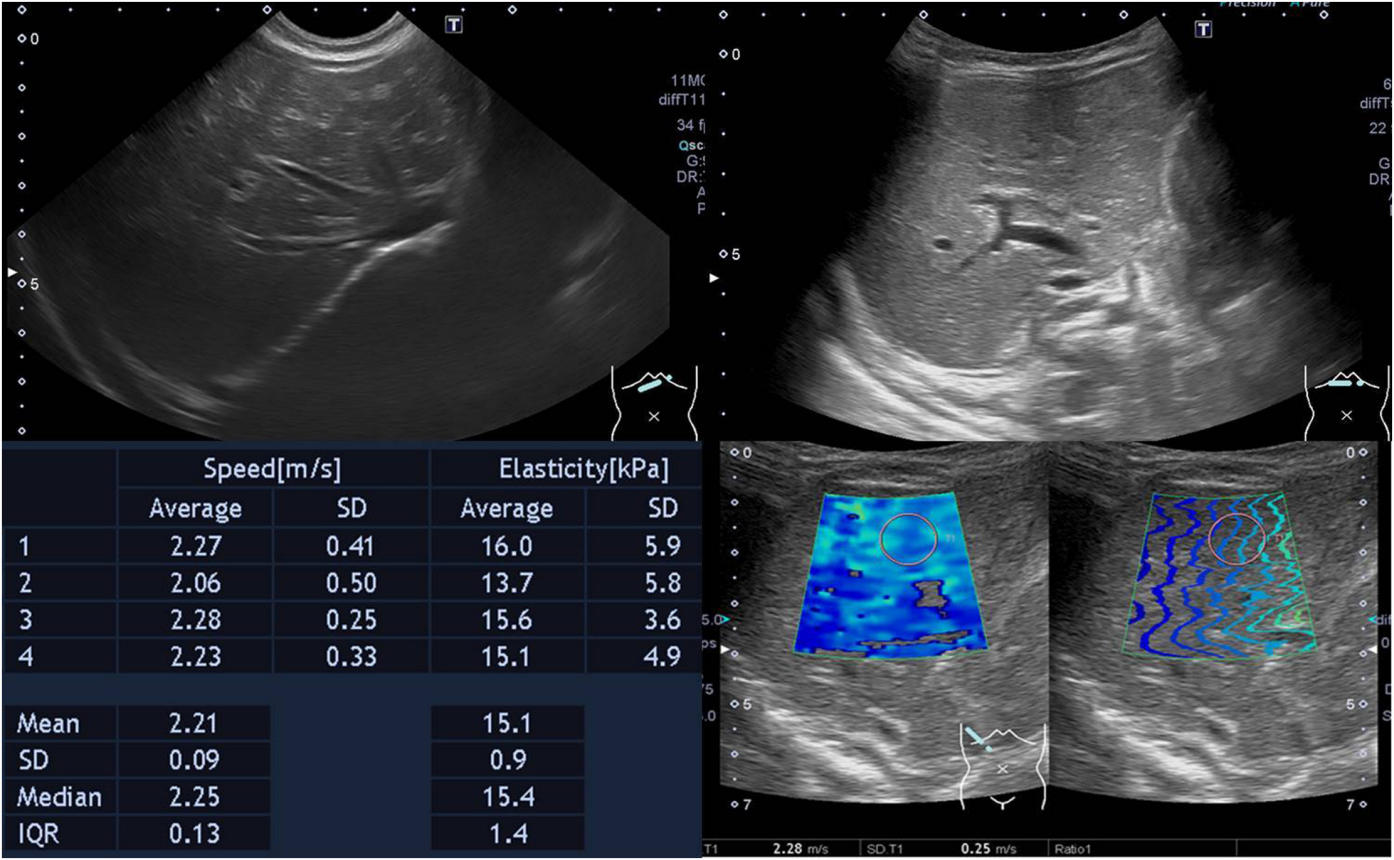
Abdominal sonography at 2 months old with increased shear wave elastography (Toshiba Aplio 500).

**FIGURE 2:**
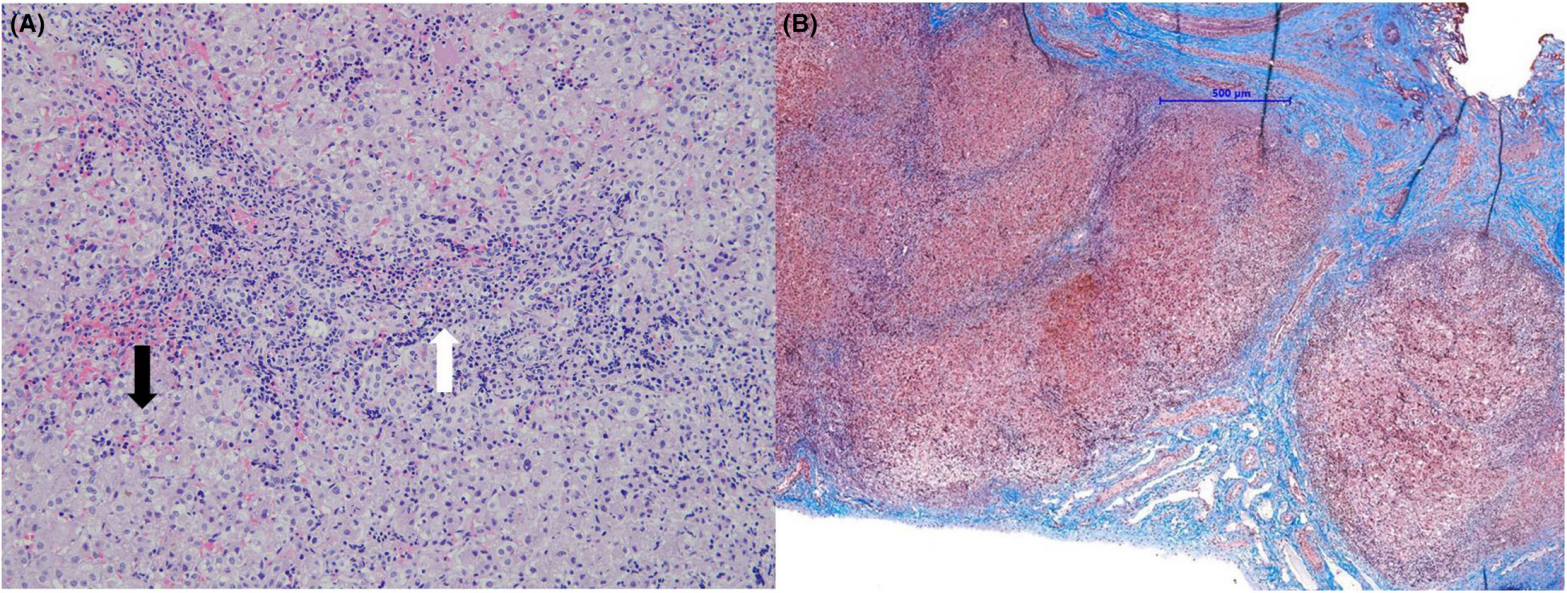
Histopathologic findings with (A) ductule proliferation (white arrow) and small fatty droplets deposition (black arrow), H&E stain 200X (B) Liver firorsis with nodular change with Masson‐Trichrome staining, 20X.

**FIGURE 3:**
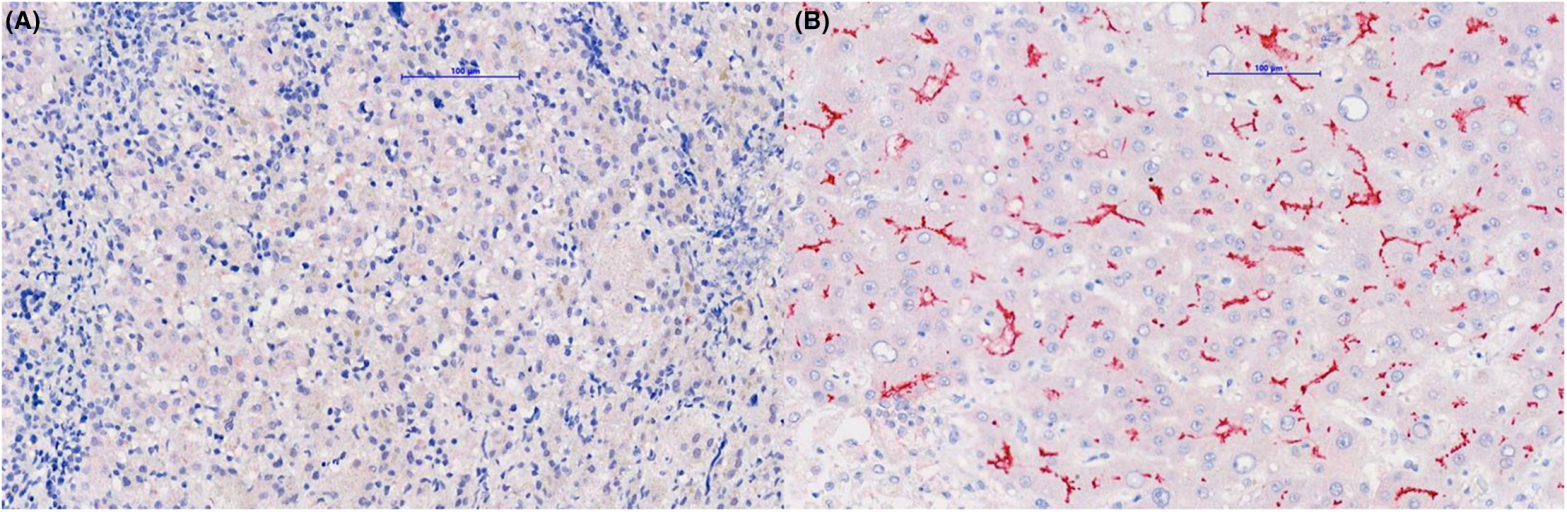
Liver immunostatin with BSEP (Santa Cruz Biotechnology, sc‐74,500) (A) totally loss of canalicular staining of BSEP in our patient (B) normal pattern of BSEP expression in control liver.

We did the survey for cholestasis, including viral (herpes simplex virus‐1, herpes simplex virus‐2, and cytomegalovirus) infection and thyroid function, was negative. Abdominal sonography showed coarse liver parenchyma with increased liver stiffness by shear wave elastography (average 15.1 kPa, Figure 1). Laparoscopic intraoperative cholangiogram revealed a patent common bile duct (CBD) without extrahepatic obstruction. Liver pathology showed mild giant cell transformation, microvesicular fatty change, and portal fibrosis with nodular change (Figure 2A,B). Liver immunohistochemistry showed no detectable expression of BSEP in bile canaliculi in our patient (Figure 3). Subsequent data still revealed low GGT cholestasis and prolonged INR despite the prescription of daily oral ursodeoxycholic acid (20 mg/kg/day) and weekly intramuscular vitamin K1 (1 mg) injections for 2 months in the follow‐up visits. Finally, whole exome sequencing (WES) showed a novel heterozygous *NR1H4* stop‐gain mutation in exon 8 (c.788C > A, p.Ser263*). This mutation is predicted as likely pathogenic by the American College of Medical Genetics and Genomics (ACMG) and is present at an allelic frequency of 0.01087% in Southeast Asian populations. We further performed array‐based comparative genomic hybridization (a‐CGH) using an Agilent SurePrint G3 human CGH microarray 1 × 1 M (G4447A, Agilent Technologies, USA) and an Agilent SureTag DNA Labeling Kit (#5190–3400, Agilent Technologies, USA) following the manufacturer's instructions to determine the copy number variation of the other *NR1H4* allele. The array contained 44 probes distributed over the *NR1H4* gene. The results revealed no small copy number variant in *NR1H4* or other *PFIC* genes.

### 2.3 Outcome and follow‐up

Her parents refused Sanger Sequencing to confirm the hereditary origin of the mutation. Subsequent laboratory data showed progressive cholestasis, coagulopathy, hyperammonemia, and high alpha‐fetoprotein levels (Table 1). She developed hepatic encephalopathy while waiting for liver transplantation. She expired at 9 months of age.

## 3 DISCUSSION

*NR1H4* mutations cause different cholestatic diseases. Heterozygous *NR1H4* variants (1G > T, M1V, W80R, M173T) cause a milder form of cholestasis such as drug induced cholestasis and intrahepatic cholestasis of pregnancy.^3^, ^4^ Only 10 cases of *NR1H4*‐related neonatal progressive cholestasis have been reported in the literature. Most of them were homozygous or compound heterozygous mutations and only one case with a heterozygous mutation had been reported (Table 2).^1^, ^2^, ^5^, ^6^, ^7^, ^8^ Our patient presented a typical phenotype of PFIC‐5 including low GGT cholestasis, vitamin K‐independent coagulopathy, high AFP levels, and rapid progression to liver failure. WES results showed a heterozygous *NR1H4* stop‐gain mutation in exon 8 (c.788C > A, p.Ser263*). Gomez‐Ospina et al. reported that one patient had a 31.7 kb copy number loss in the second allele of *NR1H4*.^6^ We further performed aCGH analysis for the detection of small copy number variant in *NR1H4* and other *PFIC* genes (*ATP8B1*, *ABCB11*, *ABCB4*, *TJP2*, *SLC51A*, *USP53*, *KIF12*, *ZFYVE19*, *MYO5B*, *SEMA7A*, and *VPS33B*), which showed negative results. The correlation between genotype and phenotype in PFIC‐5 is unclear^5^ and variable disease presentations and different age of disease onset are described in the literature.^5^, ^6^ Thus, to determine whether a heterozygous mutation is sufficient to induce a rapid progression to liver failure or the mutation of another allele may be beyond current detection methods, more studies are needed to clarify this issue.

**TABLE 2:** The *NR1H4* mutation related cholestasis in literature reviews.

Author	Year	Age at presentation	Symptoms	Mutation	Prognosis
Chen et al.^2^	2012	Infant period	Normal GGT cholestasis, cirrhosis	Heterozygous termination codon mutation of *NR1H4* R176X	Progressive cholestasis, liver cirrhosis and ascites
Gomez‐Ospina et al.^6^	2016	P1: 2 weeks P2: 2 weeks P3: 6 weeks P4: Birth	Patient1, 2, 3: cholestasis P4: neonatal cholestasis, ascites, pleural effusions and intraventricular hemorrhage at birth	P1, 2: homozygous c.526C > T (p.R176*) mutation in *NR1H4* P3, 4: homozygous in‐frame insertion variant c.419_420insAAA (p.Tyr139_Asn140insLys) in *NR1H4* and 31.7 kb deletion within *NR1H4* gene	P1 and P2 post‐liver transplantation; P3 died awaiting transplantation (8 months old); P4: died at 5 weeks old from aortic thrombus
Chen et al.^1^	2018	Neonate	Neonatal onset low GGT cholestasis; progressive coagulopathy ; Liver cirrhosis and splenomegaly	Compound heterozygous mutations in the *NR1H4* gene, c.447_448insA(p.Asn150LysfsTer6) and c.1099C > T (p.Arg367Ter)	Die of sepsis at age of 5 months
Himes et al.^5^	2020	P1: 16 months P2: 3 weeks P3: 1 weeks	P1: cholestasis, Splenomegaly P2, 3: Cholestasis, poor growth, pleural effusion	P1: homozygous pathogenic variant in NR1H4 (c.526C > T, p.R176X) P3: homozygous, out of‐frame insertion, in NR1H4 (c.276dupT, p.P93Sfs*4).	P1 liver transplantation, doing well 6 years post transplantation P2 died at 8 months old due to multiorgan failure P3 died at 7 months old due to liver failure
Czubkowski et al.^7^	2021	5 weeks	Normal GGT cholestasis, coagulopathy	Homozygous nonsense mutation in *NR1H4* of c.547C > T, p.Arg183Ter	Liver transplantation at 8 months old; died at 20 months old due to sepsis
Sophia Giang et al^8^	2021	6 weeks	Normal GGT cholestasis, Coagulopathy, failure to thrive, hepatomegaly	Homozygous mutation in *NR1H*4 of c.911dupA	Liver transplantation at 3 months old, doing well on following up
Present case	2023	2 months	Low GGT cholestasis, coagulopathy, liver fibrosis	Heterozygous *NR1H4* c.788C > A, p.Ser263* mutation	Died at age of 9 months old due to hepatic failure

Abbreviation: P, patient.

The liver histopathological exams in patient with PFIC‐5 show non‐specific findings including inflammatory cells infiltration, ductular reaction and steatosis. Early micronodular cirrhosis and fibrosis were observed in the first liver biopsy of patients with PFIC‐5.^5^, ^7^ In the present case, absent BSEP expression, portal fibrosis, and nodular changes in liver tissue were noted in liver pathology at 2 months of age. In addition, we performed the liver shear wave which showed significantly increased liver stiffness in sonography (average: 15.1 kPa, normal: 4.63 ± 0.6 kPa).^9^ Our liver shear wave findings are compatible with the liver fibrosis and nodular change observed in pathology and it suggested that fibrotic change likely began early in the progression of the disease.

Early onset vitamin K‐independent coagulopathy is the main feature of PFIC‐5 because FXR is involved in the synthesis of coagulation factors.^6^, ^10^ Typical laboratory results and early changes in liver stiffness as shown by shear wave elastography can help to take this rare disease into differential diagnosis, because early diagnosis and timely liver transplantation are the currently available treatment.^3^

This study has some limitations. First there was no FXR immunostaining on the liver pathology. However, we had liver histopathological findings and BSEP staining compatible with previous reports on the patients with PFIC‐5. Second, the heterozygous mutation in *NR1H4* was not compatible with the inheritance pattern of the gene, although we did not find any copy number variant in the other allele using aCGH. However pathogenic noncoding variants in regulatory and promotor regions, which may affect protein folding and gene regulations may not have been detected by aCGH and WES. It is important for the parents' genetic counseling to consider these issues before the next pregnancy.

In summary, PFIC‐5 is a low GGT neonatal cholestatic disease with key features of vitamin K independent coagulopathy and rapid progression of liver cirrhosis which can be early detected by noninvasive shear wave elastography. Genetic testing can help us to diagnose this rare and serious disease and provide the appropriate treatment.

## AUTHOR CONTRIBUTIONS

**Chiao‐Yu Yang:** Conceptualization; data curation; writing – original draft; writing – review and editing. **Hung‐Wen Tsai:** Data curation; investigation; methodology. **Yen‐Yin Chou:** Data curation; investigation; methodology; writing – review and editing. **Yao‐Jong Yang:** Conceptualization; resources; supervision; writing – review and editing.

## FUNDING STATEMENT

The authors declare that they have not received any funding for this study.

## CONFLICT OF INTEREST STATEMENT

All authors have no conflict of interest including the writing of the report and the decision to submit the paper for publication.

## CONSENT

We have obtained the Informed consent from the patient's parents to publish this report in accordance with the journal's patient consent policy.

## Data Availability

The authors declare that the data supporting the findings of this study are available within the article and the supplementary files.
